# Leave‐One‐Chromosome‐Out Association Testing Decodes Uncapping Behaviour in Honeybees During *Varroa destructor* Infestation

**DOI:** 10.1002/vms3.71101

**Published:** 2026-07-23

**Authors:** Peymaneh Davoodi, Mohammad Razmkabir

**Affiliations:** ^1^ Department of Animal Science Faculty of Agriculture University of Kurdistan Sanandaj Iran

**Keywords:** *Apis mellifera*, *Varroa destructor*, genome‐wide association study (GWAS), hygienic behaviour, mixed linear model, leave‐one‐chromosome‐out (LOCO)

## Abstract

**Background:**

Uncapping behaviour of Varroa‑infested brood cells is a critical social‑immunity mechanism in honeybees, enabling colonies to detect and remove parasitized brood. This trait reduces mite reproduction and pathogen transmission and is increasingly recognized as a valuable phenotypic marker for breeding Varroa‑resistant colonies.

**Objectives:**

This study aimed to identify robust genomic markers associated with uncapping behaviour by applying a statistically rigorous GWAS framework that overcomes the potential inflated associations and limited resolution of earlier fixed‑model approaches.

**Methods:**

Genome‑wide association analyses were performed using linear and logistic mixed models within a leave‑one‑chromosome‑out (MLM‑LOCO) design on Affymetrix 44K single‐nucleotide polymorphism (SNP) array data. This framework explicitly accounted for population structure and relatedness, thereby improving model accuracy and reducing false‑positive signals.

**Results:**

Both linear and logistic models identified seven loci each, with substantial overlap between approaches. Across analyses, three SNPs and nine genes consistently emerged as strong candidate genomic markers for uncapping behaviour in response to Varroa infestation. These genes encompass diverse biological functions, including immune defence, epithelial integrity, vesicle trafficking, sensory perception, metabolism, RNA regulation, stress response, and apoptosis, underscoring the complex molecular architecture underlying this behavioural trait.

**Conclusions:**

The MLM‑LOCO approach provided enhanced statistical power and eliminated inflated associations observed in fixed‑effect models, yielding a reliable set of candidate loci. These findings establish a solid genomic foundation for selective breeding of Varroa‑resistant colonies and contribute to long‑term strategies for sustainable apiculture and improved pollinator health.

## Graphical abstract

Using an MLM‐LOCO framework with linear and logistic mixed models, we identified three overlapping SNPs and nine candidate genes underlying honeybee's uncapping behavior against *Varroa destructor*. These robust markers provide a reliable genetic foundation for marker‐assisted breeding of Varroa‐resistant colonies, advancing future sustainable apiculture and global pollinator health.

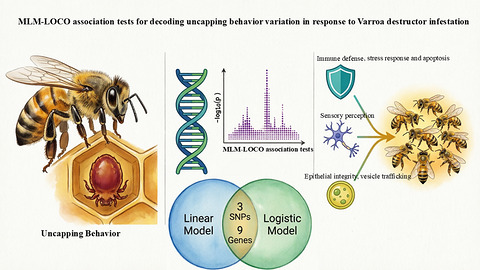

## 1 Introduction

Honeybees (*Apis mellifera*) are among the most important pollinators in agricultural and natural ecosystems, contributing substantially to global food security and biodiversity. Yet, in recent decades, honeybee populations have experienced alarming declines, with colony losses reported worldwide. Multiple stressors contribute to these declines, among these, infestations by the ectoparasitic mite *Varroa destructor* remain one of the most significant threats to colony survival (Behrens et al. 2011; O'connell et al. 2025; Guichard et al. 2020; Frazier et al. 2024). *Varroa destructor* reproduces within brood cells, feeding on the fat body and haemolymph of developing pupae and adult bees (Ramsey et al. 2019, Han et al. 2024). Beyond direct parasitism, the mite acts as an efficient vector for numerous pathogenic viruses, thereby amplifying colony morbidity and mortality. The worldwide proliferation of *Varroa* has made most unmanaged colonies unsustainable, highlighting the urgent need for effective and long‐lasting resistance strategies (Frazier et al. 2024, Guichard et al. 2020).

One promising avenue lies in the natural defence mechanisms expressed by worker bees, primarily general hygienic behaviour and Varroa‐sensitive hygiene (VSH). General hygienic behaviour is the broad ability of workers to detect and remove dead or diseased brood (Khan and Ghramh 2021, Spötter et al. 2016). VSH is a more specific, heritable form of this behaviour where bees selectively target and remove brood infested with reproducing Varroa mites (Morin and Giovenazzo 2023, Guichard et al. 2022, Spivak and Danka 2021, Mondet et al. 2015, Spötter et al. 2016). These behaviours involve distinct components, including the inspection of cells, uncapping, and the actual removal of the brood. Another related but distinct resistance mechanism is recapping, where bees inspect cells and replace the capping without removing the brood. Recapping has been shown to lower mite reproductive success (Oddie et al. 2021, Grindrod and Martin 2021, De La Mora et al. 2025, Guichard et al. 2023, X. Li et al. 2024), though recapping rates are not always directly associated with overall mite population reduction. While recapping is frequently measured in phenotypic evaluations, uncapping remains the critical initial physical step required for the actual removal of infested pupae in VSH. Therefore, uncapping behaviour can be considered a distinct, genetically influenced component of social immunity that contributes to colony resilience. However, more recent genomic research has uncovered polygenic nature of uncapping (Eynard et al. 2025, Davoodi and Razmkabir 2025). Additionally, genome‐wide association studies (GWAS) are powerful tools for dissecting complex traits, and single‐nucleotide polymorphism (SNP) arrays have successfully been applied in honeybees to identify loci associated with hygienic behaviour and Varroa‐specific defence (Guichard et al. 2021, Mustafa et al. 2024, Spötter et al. 2016, Fikere et al. 2026).

Beyond trait association tests, recent work has emphasized the complexity of behavioural genetics in social insects. Hygienic behaviour likely follows an Omnigenic or even an Omnifactorial model, in which numerous loci of small effect, distributed across the genome, contribute to trait variation (Davoodi and Razmkabir 2025). Such complexity underscores the need for integrative analytical frameworks. To control for population stratification and cryptic relatedness, which can lead to false‐positive associations, we utilized a mixed linear model (MLM) framework. Specifically, we employed the leave‐one‐chromosome‐out (LOCO) approach to estimate the genomic relationship matrix (GRM). Under the LOCO model, when testing for marker‐trait associations on a specific chromosome, the polygenic background (GRM) is calculated using all genome‐wide markers except those located on the chromosome currently being tested. This strategy prevents proximal contamination, a phenomenon where the specific marker being tested unduly influences the genomic relationship estimate, thereby increasing the statistical power to detect true associations while strictly controlling the false discovery rate, improving the robustness of GWAS signals (Zhang et al. 2010, Jiang et al. 2019). The genotype and phenotype dataset used in this study were previously reported in (Davoodi and Razmkabir 2025), where a fixed linear model (PLINK) was applied to identify candidate loci. In the present work, we extend those analyses by applying an MLM with a LOCO (MLM‑LOCO) framework implemented in GCTA to better control relatedness. We aimed to increase GWAS power, improve control of population structure and genomic relationship, and produce more reliable candidate loci for downstream functional follow‑up. Then we further interpret results in the context of social‑immunity pathways to highlight candidate genes underlying uncapping behaviour.

## 2 Materials and Methods

### 2.1 Data Resources and Breeding Design

All colonies were produced through a controlled cross‐breeding program designed to generate variation in the Varroa‐specific defence trait of uncapping mite‐infested brood cells at the Institute for Bee Research, Hohen Neuendorf (Spötter et al. 2016, Spötter et al. 2012).

### 2.2 Sampling and Phenotyping

A total of 22,000 worker bees were individually labelled with numbered tags to enable identification and behavioural tracking. Sampled workers descended from 10 distinct, labelled queens (families). Colonies were maintained in observation hives with transparent glass walls, allowing direct visual inspection of the comb. Standard husbandry protocols were applied consistently across colonies, including regulated temperature, feeding, and colony management practices. Observation hives were continuously video‐recorded using infrared cameras for seven consecutive days, enabling uninterrupted monitoring of all tagged individuals. The focal trait was the Varroa‐specific defence behaviour which is a critical initial step in the broader hygienic behaviour that worker bees use to defend against brood pests and diseases. Because the exact status of each cell within the observation area was known (artificially infested, manipulation control, or untreated), only the uncapping of the specifically infested cells was scored as the focal trait. Uncapping of the control cells was excluded from this specific metric to prevent false positives. Phenotypes were retrospectively assigned to cases and controls based on recorded behaviour, following predefined definitions and quality‐control (QC) criteria (Spötter et al. 2016). Sampling was conducted across two consecutive years: in year one (label A871), 91 bees were genotyped, and in year two (label A1173), 128 bees were genotyped (Spötter et al. 2012). The two definitions of uncapping behaviour in worker bees were used. For the linear analysis, the phenotype was the continuous count of uncapped brood cells (ranging from 0 to 4) within a specific observation period. For the binary analysis, the phenotype was derived directly from this count data by categorizing the bees into two distinct groups: bees that uncapped zero cells were assigned a value of 0 (uncapping‐negative), and bees that uncapped one or more cells (1 to 4) were assigned a value of 1 (uncapping‐positive). The continuous count was not used as a covariate in the binary model; rather, it was dichotomized to form the binary trait (Spötter et al. 2012, 2016; Davoodi and Razmkabir 2025).

### 2.3 Data Quality Control

Overall, 44,000 SNPs were considered for the GWAS construction. During the QC process, a total of 29,841 variants and 219 bee samples (females) were analysed (Spötter et al. 2012). Of these, 29 samples and 1254 variants were excluded due to missing genotyping data, 1408 variants were removed following the Hardy–Weinberg exact test, and 12,082 variants were excluded based on the minor allele frequency threshold. Ultimately, 15,097 variants and 190 individuals successfully passed the filters and QC and were found to be polymorphic in our tested populations.

### 2.4 Permission to Reuse and Copyright

The datasets analysed in this study are publicly available through the Dryad Digital Repository at https://datadryad.org/dataset/ https://doi.org/10.5061/dryad.8635cs4h. We made use of these openly accessible data and have fully acknowledged and cited the source publications in accordance with copyright and reuse policies.

### 2.5 LD Score Analysis

Linkage disequilibrium score (LD score) quantifies the amount of genetic correlation between variants in a given genomic region. The LD score for a specific SNP is calculated by summing the squared correlation coefficients (*r* ^2^) between that SNP and all other SNPs within a defined region of 1000 base window. LD Score of 0 represents no detectable linkage disequilibrium, meaning the variant is independent and not correlated with nearby SNPs. A higher LD score for an SNP indicates that it is in LD with other SNPs, meaning it is likely tagging a larger number of genetic variants (Yang et al. 2011).

### 2.6 Genome‐Wide Association Study

GWASs on linear and binomial definition of uncapping trait in worker bees were performed using the GCTA software, applying the GCTA‐LOCO linear mixed model (Yang et al. 2011). This approach uses the standard linear mixed model by including the additive effect of each SNP individually. Specifically, the following model was used for GWAS:

(1) y=μ+Xb+Za+gs∗adds+e

where *𝐲* is the uncapping phenotype, *𝛍* is the overall mean, *𝐗𝐛* is the matrix and its coefficients of fixed effects (queen, year, and PC effects), *𝐙𝐚* is the matrix and its coefficient of random effects of polygenic effects derived from genomic relationship matrix (GRM), 𝒈**s** is the genotype vector for **s**th SNP, and 𝒂𝒅𝒅**s** is the additive effect of the **s**th SNP, and *e* is the residual of the models. The GRM is computed by excluding SNPs located on the same chromosome as the candidate SNP (LOCO), reducing confounding effects caused by local linkage disequilibrium (LD) (Davoodi et al. 2022). Significance thresholds for SNPs were determined using relaxed threshold of *p* value 0.001 to identify potentially relevant effects. Manhattan plots were generated using the qqman package in R (v2024.09.0). The genomic data were subsequently analysed to assess LD pattern. To define candidate genomic regions surrounding the significant genomic signals, a 200k bp window upstream and downstream of the significant SNPs was searched (Donthu et al. 2024).

### 2.7 Proportion of Variance Explained

The proportion of variance explained (PVE) calculation was utilized to estimate the contribution of significant SNPs identified through GWAS to the phenotypic variance of the uncapping trait in worker bees. This was determined using the formula established by Shim et al. (2015).

(2) $$\begin{array}{ccc} & & \mathrm{PVE}=\\ & & \frac{\left(2*\left(\beta^{2}\right)*\mathrm{MAF}*\left(1\;-\;\mathrm{MAF}\right)\right)}{2*\left(\beta^{2}\right)*\mathrm{MAF}*\left(1\;-\;\mathrm{MAF}\right))+{\left(SE_{\beta }\right)}^{2}*2*N*\mathrm{MAF}*\left(1\;-\;\mathrm{MAF}\right)}\; \end{array}$$

where 𝛽 represents the estimated effect size of the SNP, MAF is the minor allele frequency, SE_𝛽_ is the standard error of the effect size, and *N* is the sample size.

## 3 Results

### 3.1 Data Quality Control

#### 3.1.1 IBS Matrix Heatmap Analysis Before and After QC

**FIGURE 1:**
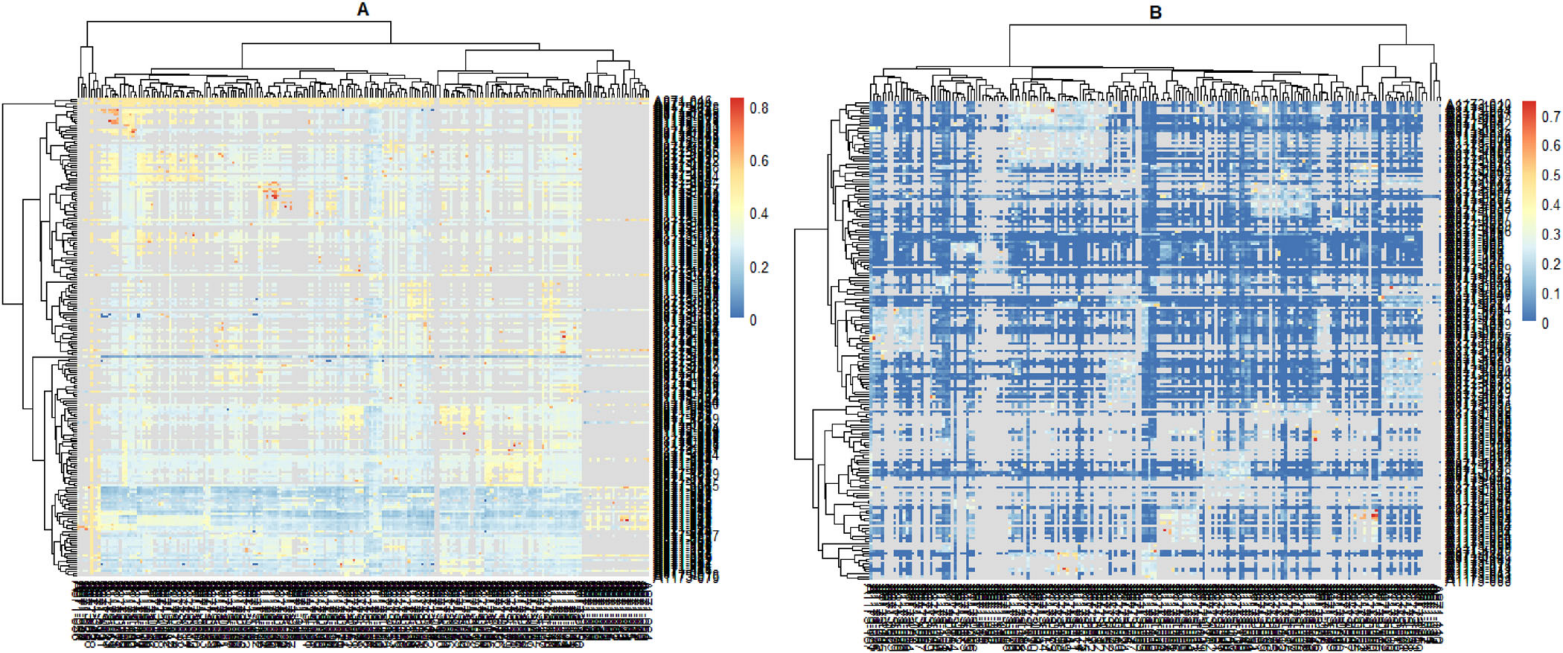
Heatmap of the squared format of the IBS Matrix. Left: raw data and right: after outlier deletion.

The IBS heatmaps (Figure 1) illustrate how QC improved the resolution of population genetic structure. Raw data showed scattered high‐similarity signals, likely due to relatedness and stratification, resulting in noisy patterns without clear clustering. After removing outliers and low‐quality samples, the IBS matrix revealed distinct block‐diagonal structures and coherent dendrograms, indicating genetically homogeneous clusters. These results emphasize the importance of rigorous filtering for accurately defining population substructure in low‐quality datasets.

### 3.2 Allele Frequency and LD Score

**FIGURE 2:**
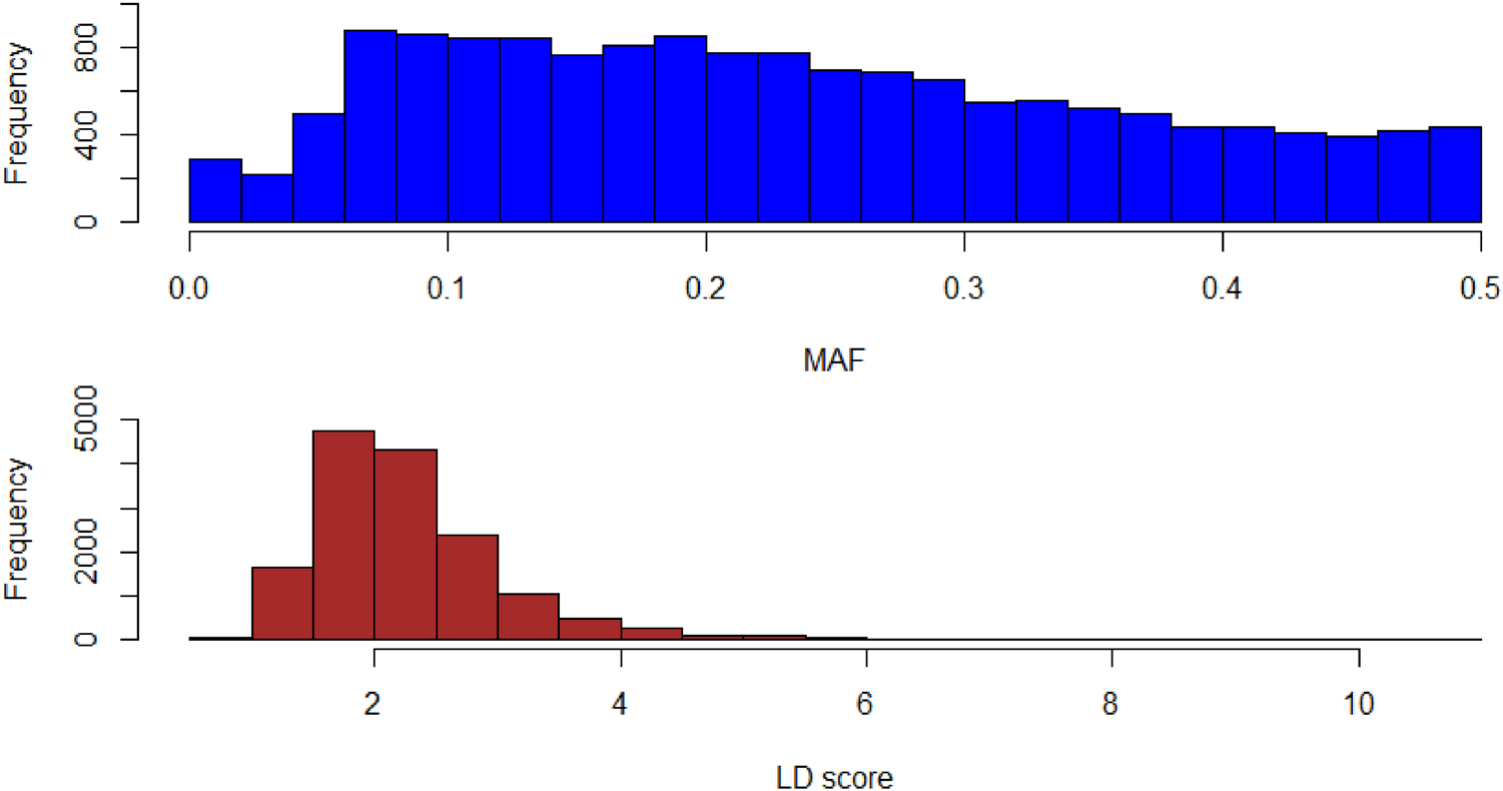
Top: allele frequency; bottom: LD score frequency in data.

The histogram of allele frequency shows a uniform scatter in the frequencies from 0 to 0.5 in the studied SNPs after QC. A local peak at a frequency of around 0.1 is also observed among the studied SNPs (Figure 2, top). The histogram of LD scores is shown in Figure 2 (bottom). In the present study, the maximum value is 10, and the peak is seen at 2. LD score analysis revealed generally low LD, with each SNP tagging fewer than 10 variants, limiting GWAS resolution. Common variants are easier to detect but typically have modest effects.

### 3.3 Quality Control of Results

**FIGURE 3:**
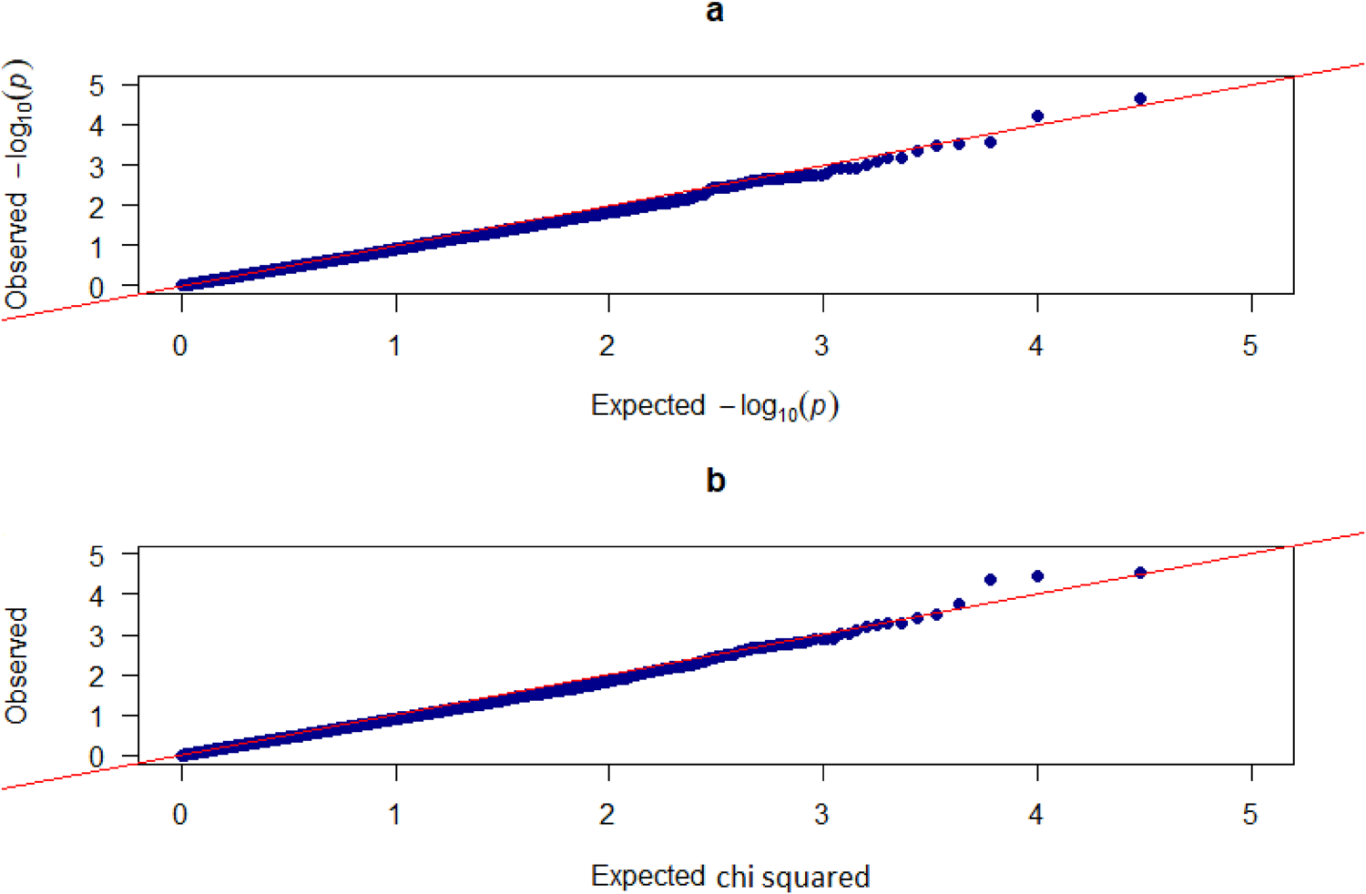
QQ plot of *p* values extracted from GWASs: (a) for linear GWAS; (b) for logistic GWAS. Diagonal red line denotes expected distribution.

The quantile–quantile plot (QQ plot) in GWAS is used to assess whether the distribution of observed *p* values aligns with the expected null distribution. It helps detect inflation or bias in association results which approves good power of test in both binary and ordinal definition of uncapping behaviour in worker bees (Figure 3).

### 3.4 Genome‐Wide Association Scenarios

**FIGURE 4:**
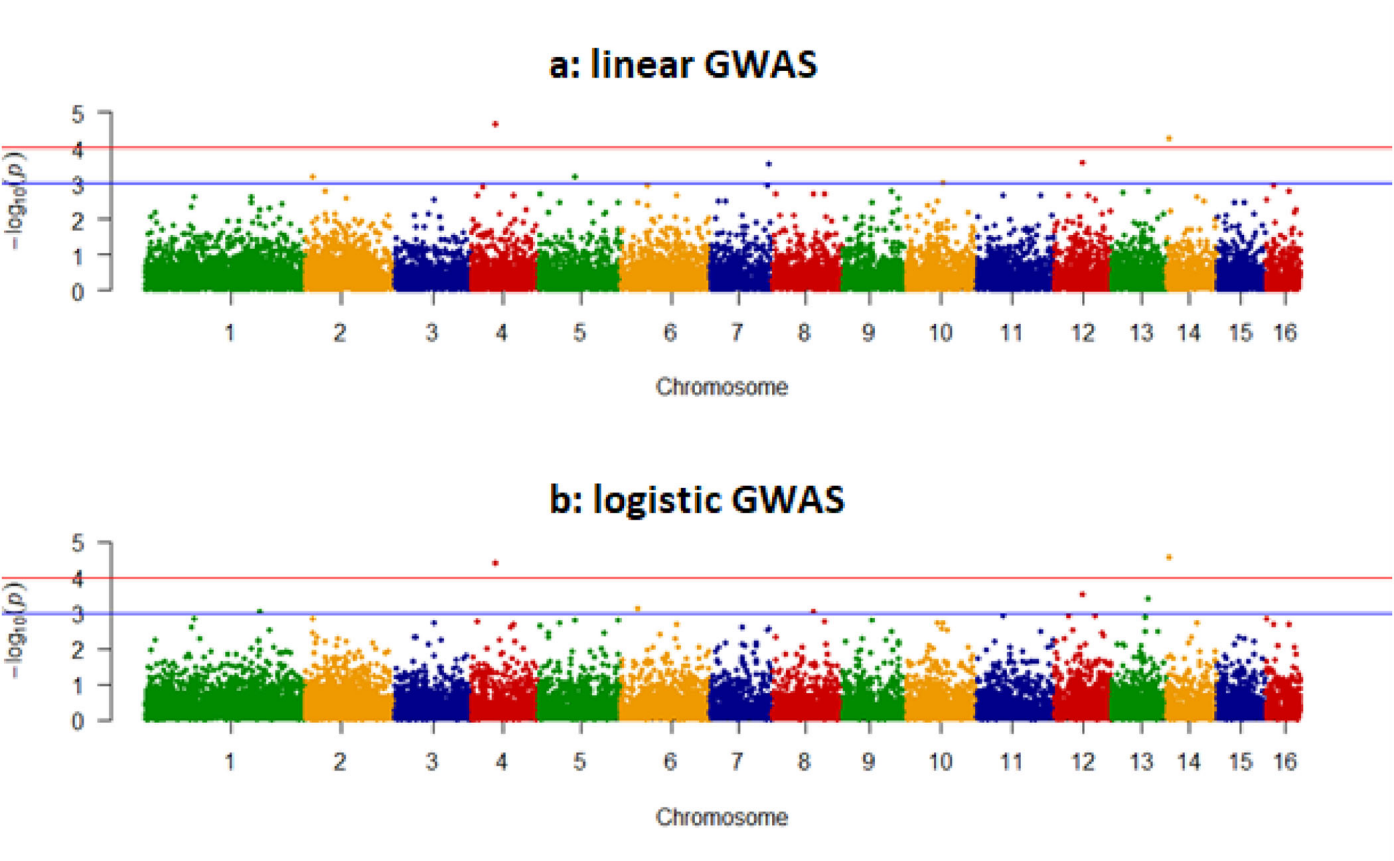
Manhattan plots to visualize the results of testing about 15,000 of genetic variants (SNPs) for association with a trait of uncapping brood cells. X‐axis: the physical position of SNPs along the chromosomes. The chromosomes are usually arranged sequentially. Y‐axis: the negative logarithm (base 10) of the *p* value or chi‐square value for each SNP's association with the trait of interest. Significance threshold: Horizontal lines indicate a significance and suggestive threshold. SNPs that fall above this line are considered statistically associated.

In linear GWAS, seven SNP markers of *AMB‐00377381*, *AMB‐00333896*, *AMB‐00037196*, *AMB‐00230239*, *AMB‐00147227*, *AMB‐00732866*, and *AMB‐00017454*, located in 4, 14, 12, 7, 2, 5, and 10 chromosomes, respectively (Figure 4a). On the other hand, in the logistic GWAS, seven SNP markers of *AMB‐00333896*, *AMB‐00377381*, *AMB‐00037196*, *AMB‐00491306*, *AMB‐00893206*, *AMB‐00901806*, and *AMB‐00794542* were positioned on chromosomes 14, 4, 12, 13, 6, 8, and 1, which are defined as top‐associated SNPs with uncapping behaviour in this study, respectively (Figure 4b).

Table 1 presents the findings of a genetic association analysis. Each row corresponds to a unique statistical model used to evaluate the association of a specific SNP with the trait of interest. The Gene column identifies the nearby gene(s) associated with each SNP, providing insights into potential biological significance.

**TABLE 1:** Summary results of genome‐wide associations and gene annotation in two models with details.

CHR	SNP	Model	BP	A1	A2	Freq	beta/OR	se	*p* value	PEV	Gene
4	*AMB‐00377381*	Linear	3906138	B	A	0.0625	−1.658	0.391	2.24E‐05	0.00179	*LOC727092, LOC102654745*
14	*AMB‐00333896*	Linear	392151	A	B	0.0693	−1.311	0.326	5.85E‐05	0.00161	*LOC100577335, LOC100577295*
12	*AMB‐00037196*	Linear	4569075	A	B	0.0710	−1.418	0.390	2.76E‐04	0.00132	*LOC408803, LOC102655281, timeout, Mir6054, Cpr97Ea, LOC411023*
7	*AMB‐00230239*	Linear	9371021	A	B	0.2526	0.729	0.202	3.09E‐04	0.00130	*LOC100577385, Tango9*
2	*AMB‐00147227*	Linear	1175695	A	B	0.0869	1.227	0.361	6.79E‐04	0.00115	*Vps13, LOC100576831*
5	*AMB‐00732866*	Linear	5889718	B	A	0.1000	0.858	0.253	6.84E‐04	0.00115	*LOC113219138, LOC551667, bigmax, LOC102655407*
10	*AMB‐00017454*	Linear	6048547	B	A	0.0500	−1.264	0.384	9.99E‐04	0.00108	*Or19, LOC100577938, LOC409427, Dhrs4, LOC10057764, LOC100577715*
14	*AMB‐00333896*	Logistic	392151	A	B	0.0694	−0.412	0.099	2.93E‐05	0.00174	*LOC100577335, LOC100577295*
4	*AMB‐00377381*	Logistic	3906138	B	A	0.0625	−0.488	0.119	4.23E‐05	0.00167	*LOC727092, LOC727285*
12	*AMB‐00037196*	Logistic	4569075	A	B	0.0710	−0.427	0.118	3.08E‐04	0.00130	*LOC408803, LOC102655281, timeout, Mir6054, Cpr97Ea, LOC411023*
13	*AMB‐00491306*	Logistic	5860294	B	A	0.2237	0.224	0.063	3.97E‐04	0.00125	*LOC725477, puf, htk, LOC725568*
6	*AMB‐00893206*	Logistic	2850479	A	B	0.2869	0.214	0.063	7.69E‐04	0.00113	*LOC412741, Mir1006, LOC100576513, LOC113219176, Mir3777*
8	*AMB‐00901806*	Logistic	6780338	A	B	0.2395	−0.218	0.066	9.00E‐04	0.00110	*LOC725202, TRNAL‐AAG, LOC107965562, LOC107965565, Ask1*
1	*AMB‐00794542*	Logistic	18365179	B	A	0.2872	−0.190	0.057	9.56E‐04	0.00109	*LOC413577, LOC551280, LOC724904, LOC102655530*

*Note*: The CHR column denotes the chromosome where the SNP is located, while BP specifies the base pair position on that chromosome. A1 and A2 are the alleles being compared, with Freq indicating the frequency of the effect allele (A1) in the studied population. The beta/OR column provides either the regression coefficient or odds ratio reflecting the strength and direction of the association, and se denotes the standard error of that estimate. The *p* value column helps assess the statistical significance of the findings.

Abbreviation: PEV: proportion of variance explained by each SNP.

### 3.5 Overlapping Results

**FIGURE 5:**
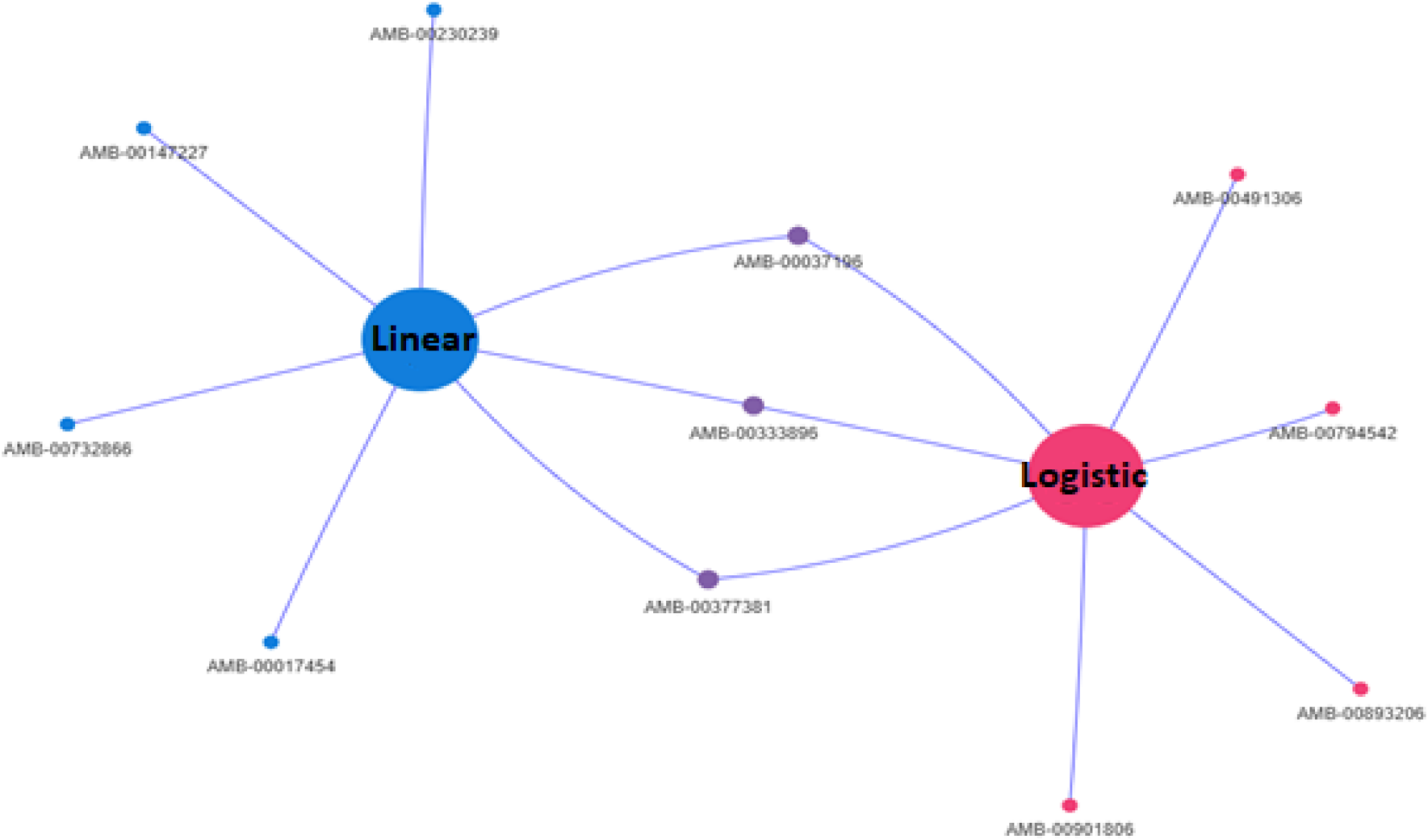
Overlapping SNPs between two MLM‐LOCO GWAS scenarios of a linear and a logistic model.

The Venn network at the SNP level (Figure 5) shows overlapping SNPs between two quantification scenarios: a linear and a logistic model, and the unique SNPs associated with each model design. Three overlapping SNPs including *AMB‐00037196*, *AMB‐00333896*, and *AMB‐00377381* were detected in both mentioned scenarios.

**FIGURE 6:**
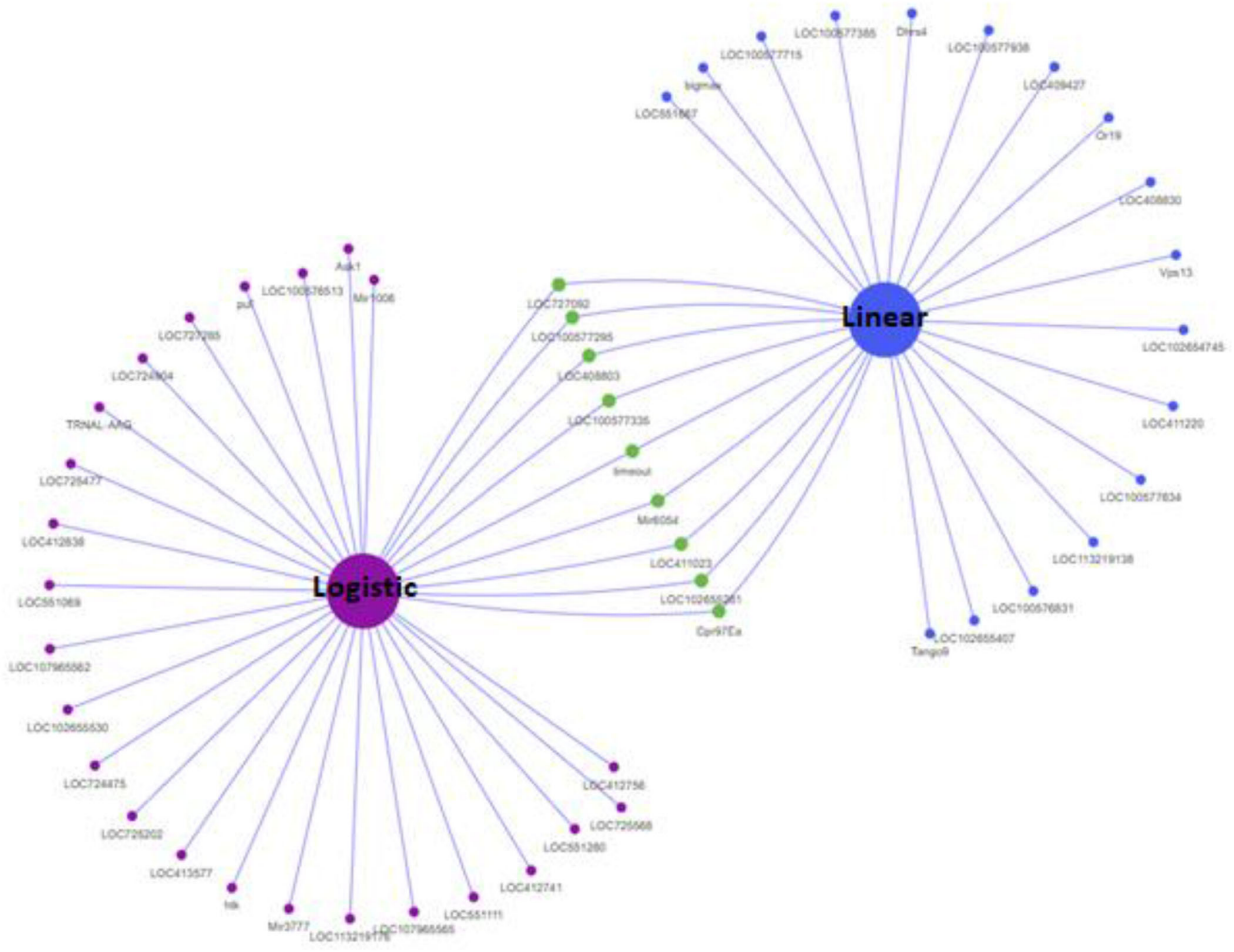
Overlapping genes between two MLM‐LOCO GWAS scenarios of a linear and a logistic model.

The Venn network at the associated gene level (Figure 6) shows overlapping genes between two scenarios of linear and logistic GWASs, and the unique associated genes in each model (17 and 24 genes). Nine overlapping genes including *LOC727092*, *LOC100577335*, *LOC100577295*, *LOC408803*, *LOC102655281*, *timeout*, *Mir6054*, *Cpr97Ea*, and *LOC411023* were detected in both linear and logistic GWASs.

## 4 Discussion

The pre‐analyses of data structure highlighted the importance of considering both LD structure and allele frequency when interpreting association signals and designing future studies (Panagiotou et al. 2010, Uffelmann et al. 2021, Visscher et al. 2017). In addition, LD scores are crucial in GWAS to account for polygenic effects and improve statistical power (Christoforou et al. 2012). The maximum value of 10 and the peak at 2 (Figure 2, bottom) indicate the condition of very diverse populations like honeybees.

Meanwhile, Spötter et al. (2016) focused on a traditional single FLM‐GWAS, while Davoodi and Razmkabir's (2025) research expanded the scope with multi‐GWAS FLM‐models (additive, dominance, and epistatic), uncovering a broader set of SNPs and candidate genes tied to immunity and stress pathways. Both studies have explored the genetic basis of Varroa resistance in honey bees through genome‐wide association approaches, but they differed in scope and methodology.

Furthermore, both studies highlighted persistent challenges in phenotype standardization, population stratification, and the power of the association test, underscoring the need for incorporating an extra efficient model (Davoodi and Razmkabir 2025, Spötter et al. 2016).

Therefore, unlike the previous works (Spötter et al. 2016, Davoodi and Razmkabir 2025), which relied on fixed linear models, the present study employed MLM‐LOCO framework with explicit control for population structure and chromosome‐specific bias. Using GCTA, we incorporated principal components and polygenic effects together with a GRM in LOCO approach for eliminating inflated associations from earlier fixed models (Yang et al. 2011).

This approach identified seven markers in the linear GWAS (four‐level quantification), seven across chromosomes 4, 14, 12, 7, 2, 5, and 10 (Figure 4a). Also, in the logistic GWAS (two‐level quantification), seven markers were detected on chromosomes 14, 4, 12, 13, 6, 8, and 1 with three SNPs consistently detected across both analyses, providing high potential evidence for their associations with uncapping behaviour in response to Varroa resistance. Therefore, this study complements the two earlier analyses conducted on the same dataset, and integrating the findings from all three investigations will provide a more comprehensive and useful understanding of the genetic basis of Varroa resistance (Davoodi and Razmkabir 2025, Spötter et al. 2016).

Although our current sample size precludes robust cross‐validation of genomic prediction accuracy, the markers and candidate genes identified here provide a critical foundation for future breeding applications by linking the genetic architecture of uncapping to specific biological mechanisms.

Among annotated genes, several protein‐coding genes with previously unknown roles, including *LOC100576831*, *LOC113219138*, *LOC551667*, *LOC100577335*, *LOC102655281*, and *LOC725477*, highlight the genomic landscape yet to be fully explored.

The discovery of noncoding microRNAs, including *Mir6054*, *Mir1006*, *Mir3777*, and *TRNAL‐AAG* (Table 1), underscores the potential importance of post‐transcriptional regulation and an additional level of molecular complexity in the genetic control of uncapping behaviour which is in agreement with X. Li et al. (2024). Because, microRNAs can act as key modulators within extensive regulatory networks, fine‐tuning social immunity pathways in honeybees. In contrast, *TRNAL‐AAG* is responsible for lysine incorporation during translation in mammalian physiology (Avcilar‐Kucukgoze and Kashina 2020).

Furthermore, *LOC727092*, a Nubbin‐like transcription factor involved in developmental regulation via the POU domain, *LOC408803* (tensin‐1) may play a role in neural or sensory processes, and *Cpr97Ea* is a cuticular protein that has a structural role in the exoskeleton (Mallick and Eleftherianos 2024). In Drosophila, POU and homeodomain proteins serve a crucial immuno‐protective function in barrier epithelia. Additionally, POU factor isoforms may function as molecular regulators, fine‐tuning the expression levels of immune effectors. These factors also play a key role in managing host–microbe interactions within the gut (Tang and Engström 2019). It is reported that Drosophila nubbin (nub) is expressed in adult midgut progenitor cells, which promotes differentiation, functions as a tumour suppressor, and helps maintain intestinal stem cell proliferation (Tang et al. 2018). The gene *LOC411023* (cadherin‐23) is also potentially linked to immune response. In addition, *Tango9* appears to regulate vesicle trafficking within the Golgi organization. Furthermore, *Tango* family genes encode transmembrane proteins that facilitate the secretion of large lipoproteins, such as chylomicrons and very low‐density lipoproteins (VLDLs), from the endoplasmic reticulum (Pfeffer 2016).

Also, the *Vps13*, a vacuolar protein sorting component, plays a role in intracellular transport. According to the previous studies on insects, *Vps* proteins play a pivotal role in regulating vesicular trafficking, essential for maintaining cellular physiology, and are often exploited by various exogenous pathogens (Z. Li and Blissard 2015, Vonk et al. 2017).

According to Havula et al. (2013) the *Bigmax* (helix‐loop‐helix‐leucine zipper transcription factor) is likely associated with growth regulation in Drosofila (Havula et al. 2013). While the *Or19*, *LOC100577938 (Or18)*, *LOC100577634 (Or17)*, *and LOC100577715 (Or15)* are odorant receptors involved in sensory perception and olfaction. Studies have shown that odorant receptors are linked to behavioural traits in social honeybee and bumblebee species (Gomez Ramirez et al. 2023, Robertson and Wanner 2006, Paoli and Galizia 2021).

In addition, *Dhrs4* functions as a dehydrogenase/reductase participating in metabolic processes, specifically retinol metabolism (Hofmann et al. 2016). *Puf* is an RNA‐binding protein that regulates mRNA stability and translation (Fischer and Olivas 2018). *Htk* is potentially a kinase‐related protein that may contribute to signalling pathways (Scheiner et al. 2003). Lastly, *Ask1* is an apoptosis signal‐regulating kinase 1 that plays a critical role in stress response and cell death (Hattori et al. 2009).

*LOC727092* encodes a nubbin‐like transcription factor with essential roles in immune protection, epithelial integrity, and gut homeostasis in *Drosophila* via POU domain regulation. Alongside the developmental and neural relevance of *LOC408803* and the structural contributions of *Cpr97Ea*, this highlights diverse gene functions. *LOC411023*, linked to immune response, together with *Tango* and *Vps13*, illustrates complex regulatory networks in vesicle trafficking and intracellular transport, critical for cellular physiology and pathogen defence. Additional genes, including odorant receptor *Or19, Or18, Or17, Or15*, metabolic enzyme *Dhrs4*, translational regulator *Puf*, *kinase Htk*, *tRNA TRNAL‐AAG*, and apoptosis signal‐regulating kinase *Ask1*, contribute to sensory perception, metabolic balance, mRNA stability, signal transduction, and stress responses. Collectively, this gene set reveals the interplay of developmental, immune, and regulatory processes, providing a foundation for functional validation and insights into their biological significance and evolutionary conservation in insects (Ma et al. 2025). These genes span diverse biological functions, from immune defence, epithelial integrity, vesicle trafficking, to growth regulation, sensory perception, metabolism, RNA regulation, and stress response, illustrating a complex network of molecular mechanisms underlying uncapping behaviour in honeybees.

Then to better understand the genetic basis of uncapping behaviour in honeybee workers, the contribution of individual SNPs to phenotypic variation using PEV estimates was quantified. Each PEV value represents (Table 1) the proportion of phenotypic variance in uncapping behaviour that's explained by an individual significant SNP (Zhu and Zhou 2020). These PVE estimates are small, ranging from 0.11% to 0.18% per SNP, which is expected for complex behavioural traits influenced by many loci and environmental factors. The summed PVE of the identified SNPs is ≈1.5%, but because SNPs are not independent, this summation should be interpreted as a descriptive summary rather than an exact partition of genetic variance. Despite their small individual effects, these markers can still be biologically informative when aggregated into polygenic scores or used as components of marker‑assisted selection, especially when combined with functional evidence of pathway enrichment and validated in independent cohorts.

A critical aspect of interpreting our findings is comparing them with previous analyses of this same foundational dataset, namely Spötter et al. (2016) and Davoodi and Razmkabir (2025). While all three studies utilize the same underlying phenotypic and genotypic data, they yield different sets of significantly associated SNPs. This divergence is primarily driven by methodological advancements in controlling for confounding factors. Spötter et al. (2016) employed a fixed linear model approach (combining Fisher's exact tests across families) on a subset of 122 cases and 122 controls, without correcting for population stratification or cryptic relatedness. This approach is susceptible to inflated false‐positive rates due to underlying family structure. Davoodi and Razmkabir (2025) expanded the analytical scope by testing additive, dominance, and epistatic models, but still relied on fixed linear models (FLMs) that may not fully account for residual population stratification or proximal contamination.

In contrast, the present study implements an MLM‐LOCO framework. By explicitly modelling the GRM and excluding the tested chromosome from the kinship estimate, our approach strictly controls for cryptic relatedness and prevents proximal contamination. This stringent control eliminates the inflated associations observed in earlier fixed models, which explains why some previously reported SNPs did not reach significance here, while new, more robust signals emerged. Furthermore, differences in QC thresholds, exact phenotype definitions (quantitative counts vs. binary case‐control), and the inclusion of specific covariates (queen, year, and principal components) naturally shift statistical power to different subsets of loci within this highly polygenic trait. Therefore, rather than contradicting previous work, our findings represent a methodological refinement that filters out stratification artefacts to highlight the most reliable candidate loci for Varroa resistance.

Collectively, this gene set reveals the intricate interplay of developmental, immune, sensory, and regulatory processes underlying the execution of uncapping behaviour. From a breeding perspective, these biologically validated targets offer immediate utility for marker‐assisted selection (MAS). While complex behavioural traits are highly polygenic, the major‐effect markers or highly significant SNPs identified in this study can be incorporated into selective breeding indices to enrich the population for uncapping behaviour. Furthermore, these validated markers serve as prime candidates for future genomic selection (GS) programs. As breeding programs scale up and genotype larger populations, the markers identified here can be used to build robust training populations. The biological validation of these targets ensures that the genomic estimated breeding values (GEBVs) in future GS models are anchored in biologically relevant variants, ultimately improving the accuracy and precision of predicting uncapping behaviour and accelerating the development of Varroa‐resistant honey bee colonies.

## Author Contributions

**Mohammad Razmkabir**: funding acquisition, reviewing. **Peymaneh Davoodi**: conceptualization, investigation, funding acquisition, writing – original draft, methodology, validation, visualization, formal analysis, software, data curation.

## Funding

Financial support was provided by the Vice President for Research and Innovation, University of Kurdistan, post‐doctoral contract number ص/3/9/22600 (1000$ for 1 year).

## Ethics Statement

Ethical approval was not required for this study, as the research was conducted entirely through bioinformatic analyses of publicly available datasets and did not involve any live animal experiments or human subjects.

## Conflicts of Interest

The authors declare no conflicts of interest.

## Data Availability

The datasets used in this study are publicly available at Dryad repository with the following link: https://datadryad.org/dataset/doi:10.5061/dryad.8635cs4h.
